# Effects of the Crack Tip Constraint on the Fracture Assessment of an Al 5083-O Weldment for Low Temperature Applications

**DOI:** 10.3390/ma10070815

**Published:** 2017-07-18

**Authors:** Dong Hyun Moon, Jeong Yeol Park, Myung Hyun Kim

**Affiliations:** Department of Naval Architecture and Ocean Engineering, Pusan National University, Busan 46241, Korea; dhyun@pusan.ac.kr (D.H.M.); cpu2565@pusan.ac.kr (J.Y.P.)

**Keywords:** crack tip constraint, *J-Q* theory, aluminum alloy, cryogenic temperature, constraint-based fracture assessment

## Abstract

The constraint effect is the key issue in structural integrity assessments based on two parameter fracture mechanics (TPFM) to make a precise prediction of the load-bearing capacity of cracked structural components. In this study, a constraint-based failure assessment diagram (FAD) was used to assess the fracture behavior of an Al 5083-O weldment with various flaws at cryogenic temperature. The results were compared with those of BS 7910 Option 1 FAD, in terms of the maximum allowable stress. A series of fracture toughness tests were conducted with compact tension (CT) specimens at room and cryogenic temperatures. The Q parameter for the Al 5083-O weldment was evaluated to quantify the constraint level, which is the difference between the actual stress, and the Hutchinson-Rice-Rosengren (HRR) stress field near the crack tip. Nonlinear 3D finite element analysis was carried out to calculate the Q parameter at cryogenic temperature. Based on the experimental and numerical results, the influence of the constraint level correction on the allowable applied stress was investigated using a FAD methodology. The results showed that the constraint-based FAD procedure is essential to avoid an overly conservative allowable stress prediction in an Al 5083-O weldment with flaws.

## 1. Introduction

Liquefied natural gas (LNG) storage, transportation, and supply systems, such as LNG carriers (LNGC), floating storage regasification units (FSRU), and LNG fuel gas supply systems (FGSS), should be designed to ensure structural integrity under a wide range of loading conditions at cryogenic temperature through the appropriate selection of low temperature materials. Typical low temperature materials applied to LNG cargo tanks include aluminum alloys, nickel alloy steels, and stainless steels. These materials exhibit excellent mechanical properties at cryogenic temperature. Among these material candidates, aluminum alloys have many advantages, such as erosion resistance and weight mitigation.

**Figure 1:**
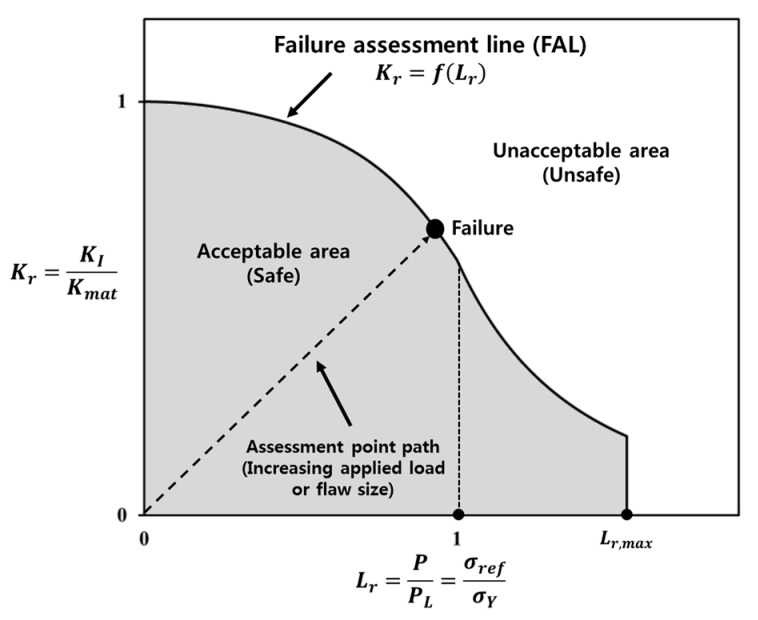
Schematic illustration of the failure assessment diagram for a structure with flaws.

Most cargo tanks for LNGC are exposed to severe loading conditions, i.e., fatigue, sloshing impact, and thermal loading. These loading conditions can often cause the failure of structural components at welded joints with flaws. Therefore, one of the most important issues in the design and construction of LNGC, is a fracture assessment of welded joints with possible flaws. Fracture assessment procedures for welded joints with flaws have been published, such as BS 7910 [1], R6 [2], and API 579 [3]. These standard procedures are based on the failure assessment diagram (FAD). In the FAD scheme, a simple geometry-independent failure assessment line (FAL) is constructed by the relationship between the fracture ratio and the applied load ratio. The fracture assessment of welded joints with a flaw is based on the relative location of the assessment point, as shown in Figure 1, with respect to the FAL. Section 2 provides further details of the FAD methodology.

**Figure 2:**
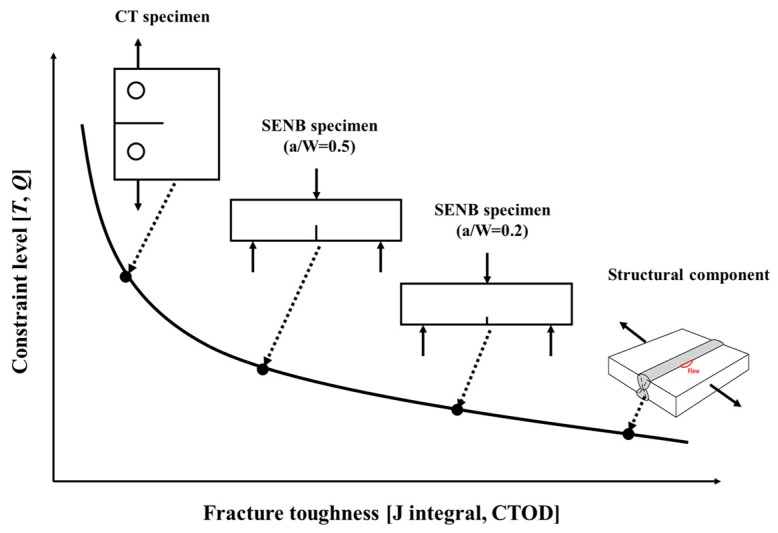
Schematic diagram of the relationship between the constraint and specimen geometry on the fracture toughness.

The application of the FAD procedure to assess the structural integrity of welded joints with flaws requires the fracture toughness values, such as crack tip opening displacement (CTOD) or J integral. In general, the fracture toughness is obtained from a fracture toughness test on a deeply cracked specimen in accordance with the established standards, such as BS 7448 [4] and ASTM E1820 [5]. These standards require that the plane–strain condition be met and guarantee high levels of stress triaxiality near the crack tip. On the other hand, flaws at the welded joints of the structural component in service are usually shallow cracks, which show low levels of stress triaxiality near the crack tip [6]. This is described as a low constraint level and leads to an increase in fracture toughness, as shown in Figure 2. In elastic–plastic fracture mechanics, it has been established that the J integral describes the crack tip stress field well under high constraint conditions. For low constraint conditions, however, the J integral alone does not express the actual stress field near the crack tip precisely. In other words, the actual stress deviates from the stress described by the J integral, and the difference between the actual stress, and the stress described by the J integral near the crack tip, is designated as the constraint level. Therefore, the structural integrity assessments of low constraint condition structural components using the fracture toughness values obtained from deeply cracked fracture specimens may be overly conservative, which may result in unnecessary maintenance time and cost [7,8]. 

In this regard, the transferability of the fracture toughness obtained from a laboratory specimen to an actual cracked structural component, becomes an important issue in structural integrity assessments based on the fracture mechanics. Extensive studies have examined the constraint effects to provide a more accurate characterization of the crack tip fields by introducing a second parameter. Several theories or methodologies for the constraint analysis of cracked structures based on two-parameter fracture mechanics (TPFM) have been proposed to quantify the constraint level, as reviewed by Cicero et al., and Zhu and Joyce [6,8]. The single-parameter fracture mechanics theory assumes that the fracture toughness obtained from laboratory specimens can be transferred to structural applications. Two-parameter approaches, however, imply that the laboratory specimen must match the constraint of the structure, i.e., the two geometries must have the same constraint level at failure in order for the respective fracture toughness values to be equal [9]. 

Two-parameter approaches based on the J-T approach [10], effectively quantify the crack tip stress fields for low constraint conditions, such as shallow cracked structures. The J-T approach has limitations, however, because the T-stress is an elastic parameter that becomes less meaningful as the plastic zone expands at the crack tip. To address this limitation, O’Dowd and Shih [11,12] studied a new second parameter, Q, for an elastic–plastic crack under small scale yielding (SSY) and large scale yielding (LSY) conditions [6]. Based on J-Q theory, Nevalainen and Dodds [13] performed 3D nonlinear finite element analyses to provide an extensive parametric evaluation of the crack front stress triaxiality for single edge notched bend (SENB) and compact tension (CT) specimens. In addition, Graba proposed closed form formulae to determine the Q-stress for SENB specimens [14] and a center cracked plate in tension (CCT) [15], with respect to the various strain hardening exponents and yield strengths. Based on these studies, recent studies have focused on the constraint, which is one of the key issues in TPFM and the structural integrity assessment, to define the constraint loss in cracked structural components. For example, Cravero and Ruggieri [16] evaluated the application of constraint-based FADs for high pressure pipelines with axial surface cracks. They indicated that constraint-based FADs have a strong geometry dependence, which is associated with crack tip constraint, than conventional FAD in BS 7910 Option 1. Jin and Wu [17] observed a similar trend. They conducted a structural integrity assessment for pressure vessel welds using constraint-based FADs, and compared their results with those obtained for BS 7910 Option 1 FAD. Their results showed that the Q parameter obtained from 2D plane strain finite element analysis decreased with increasing applied load, and the Q parameter of a deep crack is higher than that of a shallow crack at the limit load in the case of SENB specimens. On the other hand, there has been no comparative investigation discussing the effects of a constraint-based fracture assessment for the welded joints of Al 5083-O, and the conventional integrity assessment procedure at cryogenic temperature.

The purpose of this study was to investigate the application capability of the constraint-based fracture assessment procedure for the welded joints of Al 5083-O with surface flaws at cryogenic temperature. The fracture toughness tests were conducted for CT specimens at room and cryogenic temperatures. Moreover, 3D finite element analysis was used to calculate the Q parameters at the weldment for various cracks. Constraint-based FAD results from this study were examined and compared with those obtained for BS 7910 Option 1 FAD in terms of the maximum allowable stress.

## 2. Constraint-based Failure Assessment

### 2.1. The J-Q Theory

Extensive studies have been conducted to characterize the crack tip stress and strain fields based on the fracture mechanics. Hutchinson [18], and Rice and Rosengren [19], reported that the J integral characterizes the crack tip stress field in nonlinear elastic material. They assumed a power law relationship between the plastic strain and stress as

(1) $$\frac{\varepsilon }{\varepsilon_{0}}=\frac{\sigma }{\sigma_{0}}+\alpha {\left(\frac{\sigma }{\sigma_{0}}\right)}^{n},$$

where $\varepsilon_{0}=\sigma_{0}/E$ is the yield strain, $\sigma_{0}$ is the yield stress, E is the elastic modulus, α is the material constant, and n is the strain hardening exponent. Based on the power law described in Equation (1), they proposed the J-controlled crack tip stress known as the Hutchinson-Rice-Rosengren (HRR) stress field as follows:

(2) $${\left(\sigma_{ij}\right)}_{HRR}=\sigma_{0}{\left(\frac{J}{\alpha \varepsilon_{0}\sigma_{0}I_{n}r}\right)}^{\frac{1}{n+1}}{\tilde{\sigma }}_{ij}\left(n,\;\theta \right),$$

where J is the J integral, $I_{n}$ is an integration constant that depends on n, and ${\tilde{\sigma }}_{ij}$ are the dimensionless functions of n and θ. Here, r and θ are the polar coordinates centered at the crack tip with θ=0 corresponding to the line of ahead of the crack tip. 

As mentioned in Section 1, O’Dowd and Shih [11,12] carried out a series of detailed elastic–plastic finite element analyses for various geometries based on deformation plasticity theory. Using the full-field finite element analysis results, they reported that the difference field in Equation (3) between the full-field numerical solution, $\sigma_{ij}$, and the HRR stress field, ${\left(\sigma_{ij}\right)}_{HRR}$, is relatively constant, with both distance and angular position in the forward sector of the crack tip region, (|θ|≤π/2):

(3) $$\sigma_{ij}={\left(\sigma_{ij}\right)}_{HRR}+{\left(\sigma_{ij}\right)}_{Diff}.$$

The difference field corresponds approximately to a uniform hydrostatic shift of the stress field in front of the crack tip. Accordingly, they designated the amplitude of this approximate difference field by the Q parameter. Consequently, they developed the so-called the J-Q theory. Equation (3) expresses the two-term crack tip stress field as follows:

(4) $$\sigma_{ij}={\left(\sigma_{ij}\right)}_{HRR}+Q\sigma_{0}\delta_{ij},\;\text{for}\;r>J/\sigma_{0}\;\text{and}\;|\theta |\leq \pi /2,$$

where Q is a stress triaxiality parameter to reflect the hydrostatic stress level at the crack tip, and $\delta_{ij}$ is Kronecker delta [6,9]. Based on Equation (4), the Q parameter can be inferred by subtracting the HRR stress field from the actual stress field. Therefore, the Q parameter can be defined by the crack opening stress obtained from finite element analysis as follows:

(5) $$Q=\frac{{\left(\sigma_{\theta \theta }\right)}_{FEA}-\;{\left(\sigma_{\theta \theta }\right)}_{HRR}}{\sigma_{0}},\;\text{at}\;r=\frac{2J}{\sigma_{0}}\;\text{and}\;\theta =0,$$

where ${\left(\sigma_{\theta \theta }\right)}_{FEA}$ is the crack opening stress calculated by finite element analysis, and ${\left(\sigma_{\theta \theta }\right)}_{HRR}$ is the HRR stress obtained from Equation (2).

For the bending dominated specimens, such as CT and SENB specimens, various numerical results showed that the global bending moment can significantly impinge the crack tip stress field under the LSY or fully plastic conditions. Accordingly, the J-Q theory may lose its validity to describe the crack tip field for the bending geometries under large plastic deformation [6,20]. To obtain a load-independent constraint parameter, Zhu et al. [21] introduced a modified parameter, $Q^{*}$, as follows:

(6) $$\frac{\sigma_{ij}}{\sigma_{0}}={\left[\frac{J}{\alpha \varepsilon_{0}\sigma_{0}I_{n}L}\right]}^{\frac{1}{n+1}}\left[{\left(\frac{r}{L}\right)}^{-\frac{1}{n+1}}{\tilde{\sigma }}_{ij}\left(n,\;\theta \right)+Q^{*}\delta_{ij}\right],\;\text{for}\;r>J/\sigma_{0}\;\text{and}\;\left|\theta \right|\leq \pi /2,$$

where $\varepsilon_{0}=\sigma_{0}/E$ is the yield strain, $\sigma_{0}$ is the yield stress, E is elastic modulus, α is the material constant, and n is the strain hardening exponent. Here, L is the characteristic length, $I_{n}$ is an integration constant that depends on n, and ${\tilde{\sigma }}_{ij}\left(n,\;\theta \right)$ are the dimensionless stress functions. Based on Equations (2), (4), and (6), a modified constraint parameter, $Q^{*}$, is defined as

(7) $$Q^{*}={\left[\frac{J}{\alpha \varepsilon_{0}\sigma_{0}I_{n}L}\right]}^{-\frac{1}{n+1}}Q,$$

Further details on the $Q^{*}$ parameter can be found in Refs. [20,21]. In this study, the $Q^{*}$ determined by Equation (7), was considered to be the Q parameter of the CT specimen with different crack configurations, to quantify the constraint level for the Al 5083-O weldment. In the later part of this section, $Q^{*}$ is denoted by Q for a simpler description of constraint level.

### 2.2. Modification of Failure Assessment Diagram (FAD)

The present work focuses on the fracture assessment of Al 5083-O welded joints with a surface flaw based on the FAD methodology. The feature of the FAD scheme previously described is a simple geometry-independent FAL, as shown in Figure 1, and is represented by the relationship between the fracture ratio, $K_{r}$, and the applied load ratio, $L_{r}$. Accordingly, the FAL is expressed as

(8) $$K_{r}=f\left(L_{r}\right),$$

where

(9) $$K_{r}=\frac{K_{I}}{K_{mat}},$$

and

(10) $$L_{r}=\frac{P}{P_{L}}=\frac{\sigma_{ref}}{\sigma_{Y}}.$$

Here, $K_{I}$ is the applied stress intensity factor, $K_{mat}$ is the material toughness measured by the stress intensity factor, P is the applied load, and $P_{L}$ is the limit load. Alternatively, the $L_{r}$ can be defined by the ratio of a reference stress, $\sigma_{ref}$, and yield stress, $\sigma_{Y}$ [4].

On the other hand, flaws in the welded joints of structural components are often surface cracks, which have a low constraint level that contrasts significantly with the fracture toughness test specimens. Therefore, Ainsworth [22] developed a constraint-based FAD incorporating the constraint effect by quantifying the constraint in terms of $L_{r}$. The constraint level is taken into account through the normalized structural constraint parameter, β. This parameter is expressed by the Q parameter, or T-stress, as follows:

(11) $$\beta_{Q}=\frac{Q}{L_{r}}$$

(12) $$\beta_{T}=\frac{T}{\sigma_{Y}L_{r}},$$

where $\beta_{Q}$ and $\beta_{T}$ are the normalized structural constraint parameter defined from the Q parameter and *T*-stress, respectively. Both structural constraint parameters depend on the geometry, flaw size, and type of loadings [1]. To examine the constraint effect, it is essential to have a measure of not only the structural constraint parameter, but also the dependence of material toughness on the constraint. The constraint dependent toughness, $K_{mat}^{c}$, is dependent on $\beta L_{r}$ [1,22] and is related to the structural constraint by

(13) $$K_{mat}^{c}=K_{mat}\left[1+\alpha {\left(-\beta L_{r}\right)}^{k}\right],$$

where α and k are constants dependent on the material and temperature. The modified FAD can then be obtained by

(14) $$K_{r}=f\left(L_{r}\right)\left(\frac{K_{mat}^{c}}{K_{mat}}\right),$$

where $K_{mat}^{c}$ is defined by Equation (13). Substituting Equation (13) into Equation (14), the modified FAD can be expressed as

(15) $$K_{r}=f\left(L_{r}\right)\left[1+\alpha {\left(-\beta L_{r}\right)}^{k}\right].$$

**Figure 3:**
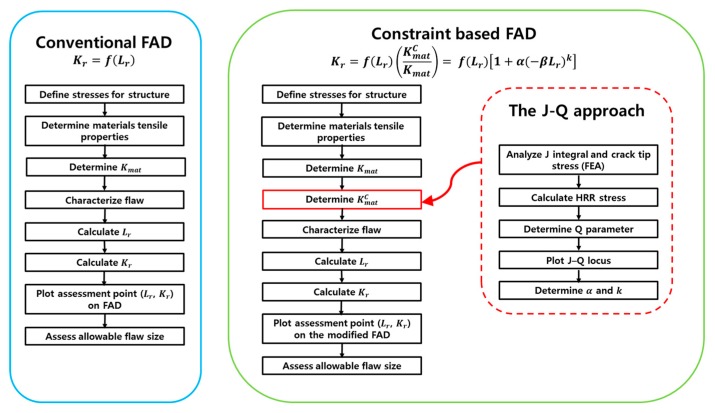
Comparison of the fracture assessment procedures between the conventional (BS 7910) and constraint-based (this study) approaches.

Note that the constraint-based fracture assessment involves the modified FAD, but retains the definition of $K_{r}$ by Equation (9). In other words, the fracture toughness obtained from the geometry with high constraint remains unchanged, but the failure assessment curve is modified by the constraint factors [1,16,17]. Figure 3 presents a flowchart for the constraint-based fracture assessment used in this study. Further details on the constraint-based structural integrity assessment procedures can be found in the BS7910 procedure [1].

## 3. Experimental Procedure

In the present work, tensile and fracture toughness tests were carried out for base and weld metals of Al 5083-O, to investigate the fracture characteristics of the welded joints for Al 5083-O. Moreover, the mechanical properties and CTOD values were measured at room and cryogenic temperatures (−163 °C).

### 3.1. Material and Tensile Test

Table 1 and Table 2 list the chemical composition of the base metal and the detailed welding conditions, respectively.

The mechanical properties of the base metal and weldments were tested according to ASTM E8 [23]. Standard round bar specimens (diameter of 12.5 mm, gage length of 50 mm, and length of the reduced section of 56 mm) were used to obtain the yield and tensile strength, elastic modulus, and elongations, at room and cryogenic temperatures. At least five specimens were tested at each material and temperature, and a total of 30 specimens were used in the tensile tests. Table 3 summarizes the mechanical properties. The maximum standard deviation for all mechanical properties shown in Table 3 is 14% of the average value. In addition, Table 3 lists the strain hardening exponent, n, and the material constant, α, of Al 5083-O obtained by the Ramberg–Osgood relationship.

### 3.2. Fracture Toughness Test

**Figure 4:**
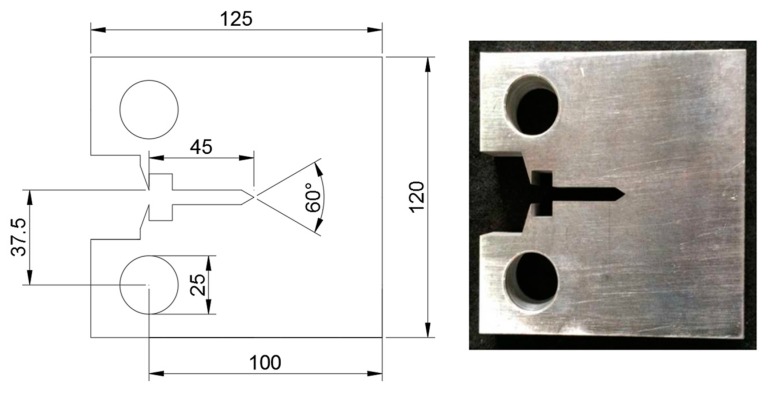
Dimensions of the compact tension (CT) specimen for the fracture toughness test (unit: mm).

Fracture toughness tests were conducted for the base metal and weldments in accordance with BS 7448 [4]. The CT specimen, shown in Figure 4, was used to obtain the fracture toughness at room and cryogenic temperatures with a specimen thickness of 25 mm. For the weldment specimen, the machined notch was fabricated parallel to the welding direction. The fatigue pre-cracking was conducted under a constant stress ratio (R = 0.1) for each specimen. The cryogenic test temperature was maintained in a cryogenic chamber equipped with a LN2 (Liquid Nitrogen) gas inlet–outlet control system. The fatigue pre-cracking and fracture toughness tests were performed with a servo hydraulic testing machine (IST-8800, INSTRON). A cryogenic clip gage capable of functioning at −190 °C was attached to each specimen to measure the crack mouth opening displacement (CMOD). The load versus CMOD curve was acquired for each test, and the CTOD values were calculated according to BS 7448 [4]. In this study, a total of 20 specimens were tested.

## 4. Numerical Procedure

**Figure 5:**
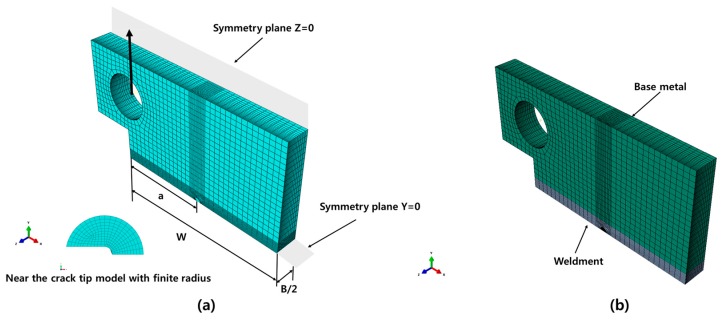
(**a**) Finite element model for the CT specimen; (**b**) assignment of the material properties (a/W = 0.5).

To investigate the constraint level of the weldment in Al 5083-O, 3D nonlinear finite element analysis was carried out to calculate the Q parameter for the CT specimens. Various ratios of a/W = 0.2, 0.3, 0.4, and 0.5, were considered in this numerical calculation. Here, a/W is the crack ligament ratio, a is the crack length, and W is the width of the specimen. Considering the symmetry in the geometry, and the loading, a quarter portion of the specimen was modeled in the finite element model. The CT specimen was modeled as a bi-material consisting of base and weld metals, with material properties assigned on the corresponding regions. The stress–strain response of the base and weld metals was used in the 3D models according to the true stress–strain relationship obtained from the tensile tests. The welding residual stress and heat-affected zone were not considered in the finite element model, as post welding heat treatment is applied after welding. The deformation theory of plasticity, and the von Mises yield criterion, were adopted in the finite element analysis [14]. To enhance the convergence of the nonlinear iterations, the mesh configuration, with a focused ring-shaped element surrounding the crack tip front with an initial small root radius, was employed. The radius of curvature at the notch tip was modeled with 2 µm, as specified in previous research [16,17]. The eight-node linear brick elements were used in the finite element model, and the mesh was refined sufficiently to obtain an adequate resolution of the stress field near the crack tip. Accordingly, the finite element model consists of 95, 520 nodes and 84, 885 elements. Figure 5 shows the finite element model of the CT specimen, corresponding the crack ligament, a/W = 0.5. The detailed mesh near the crack tip and configurations were similarly employed in all other finite element models.

## 5. Results and Discussion

### 5.1. Results of Fracture Toughness Test

**Figure 6:**
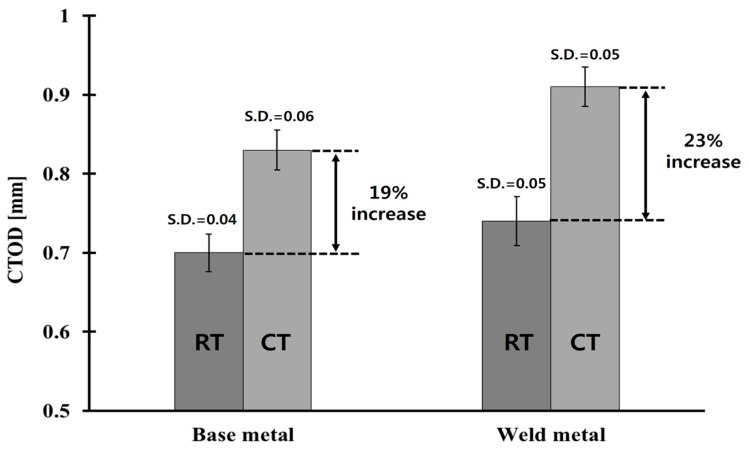
CTOD improvement ratio from room to cryogenic temperatures.

Table 4 lists the CTOD values obtained from the fracture toughness tests. At least five specimens were tested at each material and temperature, and the average values were obtained from the corresponding CTOD. The maximum standard deviation of all CTOD results is 8% of the average value. As expected, the CTOD values of both the base metal and the weldments at cryogenic temperature were higher than those measured at room temperature. This tendency is similar to previous research in [24]. In terms of the CTOD improvement ratio, as defined as the ratio of the CTOD values between cryogenic and room temperatures, the base metal and weldments of Al 5083-O exhibited similar CTOD improvement ratios, as shown in Figure 6. This suggests that the resistance to fracture of Al 5083-O, in the presence of a crack at cryogenic temperature, is higher than that at room temperature.

### 5.2. Discussion of Q Parameter in CT Specimen

**Figure 7:**
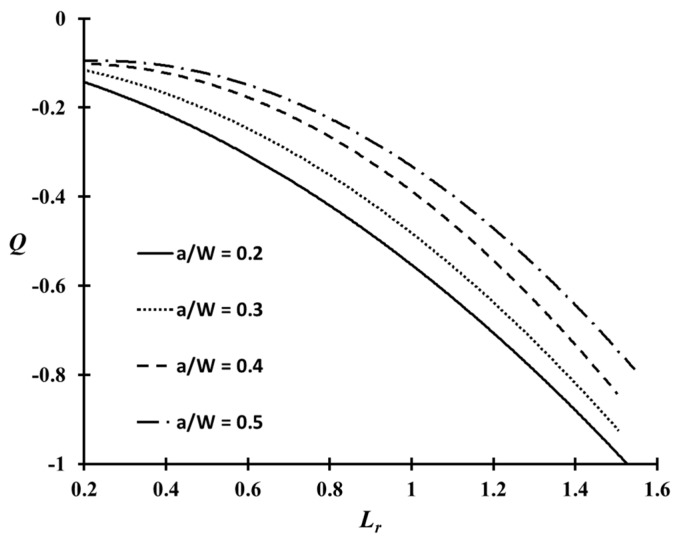
Q parameter distributions of the CT specimen for various crack configurations.

Figure 7 shows the variations of the Q parameter in the CT specimen with respect to the normalized applied load ($L_{r}$) of the weldment at cryogenic temperature. Because the variations of the Q parameter at room temperature are similar to those at cryogenic temperature, they are not shown separately. The Q values in Figure 7 decrease rapidly with increasing applied load. This tendency is consistent with previous studies of the Q parameter [14,15,16,17]. As expected, the Q parameter was influenced significantly by the crack length characterized by the a/W ratio. A shallow cracked specimen with a/W = 0.2, exhibited the lowest Q parameter distribution compared to other a/W ratios. In other words, the crack opening stress reduces under HRR stress, and the Q parameter rapidly becomes negative. Therefore, the resistance to fracture of the material in the presence of a crack is raised, due to the low stress near the crack tip. On the other hand, the Q parameter distribution for a deeply cracked specimen, such as a/W = 0.5, is relatively higher than the others, but they decrease rapidly when the applied load exceeds the limit load ($L_{r}\;$= 1). The geometry with a negative Q indicates a low constraint level near the crack tip, whereas zero or positive Q values correspond to a higher constraint level [16,17,22].

**Figure 8:**
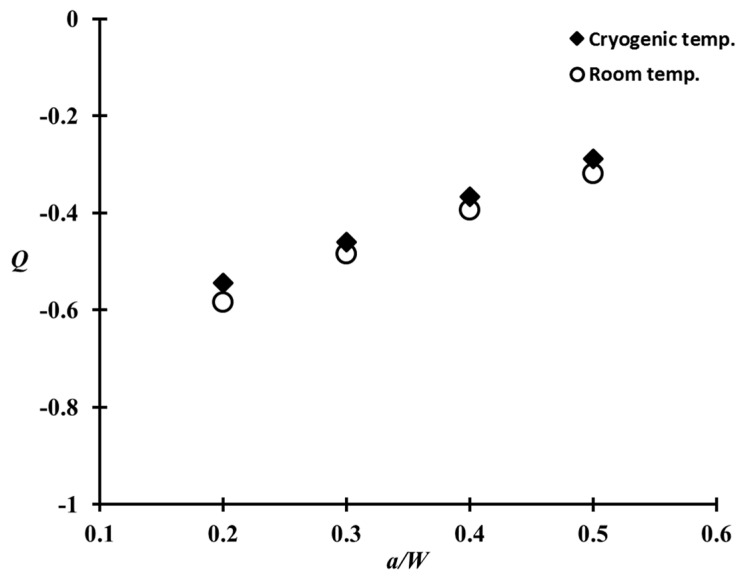
Comparison of the Q parameters for the weldment with respect to different a/W ratios at room and cryogenic temperatures.

To examine the influence of temperature in the Q parameter, the Q parameter values at room and cryogenic temperatures were compared with respect to the different a/W ratios (Figure 8). Here, the Q values correspond to the applied load equal to the limit load. The Q parameter of Al 5083-O does not vary much, typically within 7%, toward the temperature. This tendency is consistent with previous research [25].

### 5.3. Constraint-Based FAD for the Al 5083-O Weldment at Cryogenic Temperature

The purpose of this study was to investigate the constraint-based fracture assessment procedure for the welded joints of Al 5083-O with a surface flaw at cryogenic temperature. Accordingly, the functional correlation between the Q parameter and $L_{r}$ for the weldment of the CT specimen with a/W = 0.2–0.5 on the cryogenic temperature in Figure 7, was used to determine the constraint-based FAD, as defined in Equation (15).

**Figure 9:**
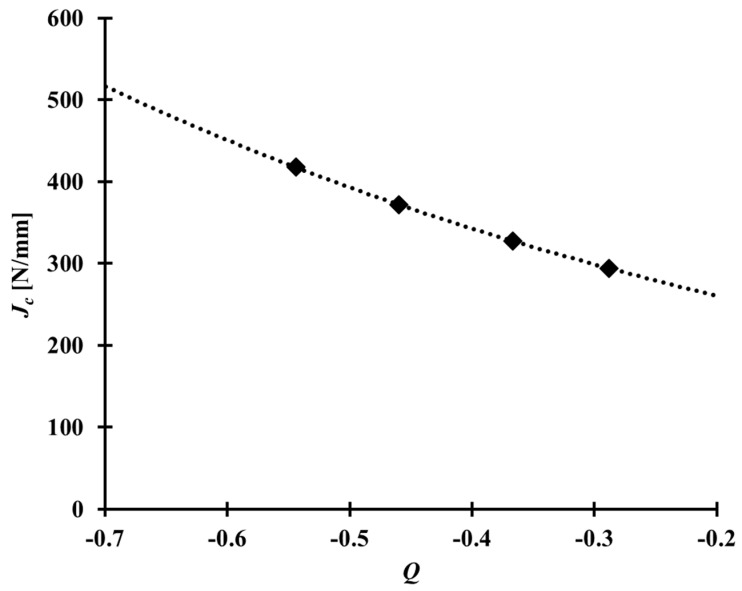
*J-Q* locus of the Al 5083-O weldment at cryogenic temperature.

In this study, the modified Ritchie-Knott-Rice (RKR) [26] local fracture criterion by Neimitz et al. [27] as the J-Q toughness locus, is used to obtain the J integral values corresponding to the Q parameter of the CT specimens with different a/W ratios. They proposed a formula to calculate the critical value of the J integral from the Q parameter, based on the experimental critical J integral value as follows [25,26]:

(16) $$J_{c}=J_{I_{c}}{\left(1-\frac{Q-Q_{ref}}{\frac{\sigma_{22}^{max}}{\sigma_{0}}-Q_{ref}}\right)}^{n+1},$$

where $J_{I_{c}}$ is the critical J integral value measured from the specimen satisfying the requirement in standards, such as BS 7448 and ASTM E1820 [4,5] as the CT specimen with a/W = 0.5; $J_{c}$ is the critical value of the J integral for the specimen with a/W in the range of 0.2–0.4, and $Q_{ref}$ is the Q parameter calculated for the reference state. Here, the reference state is associated with the specimen used to determine $J_{I_{c}}$ [27]. That is, the J integral value, $J_{I_{c}}$, is the critical J integral value for the given $Q_{ref}$ value. The maximum opening stress, $\sigma_{22}^{max}$, near the crack tip can be obtained from finite element analysis, $\sigma_{0}$ is the yield strength, and n is the strain hardening exponent of the material in consideration. Further details of the J-Q locus can be found in Refs. [26,27]. Figure 9 presents the critical J integral values determined by Equation (16) for different a/W ratios.

**Figure 10:**
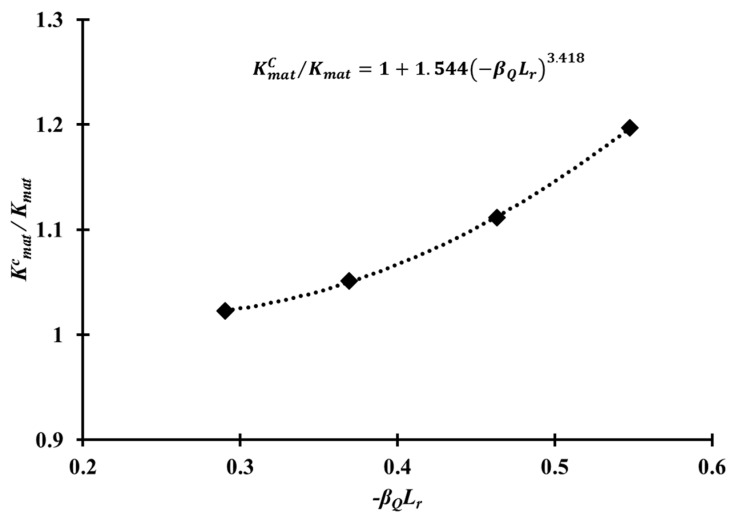
Approximation of the crack tip constraint and $K_{mat}^{c}$ to obtain α and k in Equation (13).

Based on the J-Q locus, as shown in Figure 9, and Equations (11) and (13), $K_{mat}^{c}$ was determined, and the constants α and k in Equation (13) were then obtained by regression, as shown in Figure 10. The values of α and k of the Al 5083-O weldment considered in this study, were found to be 1.544 and 3.418, respectively.

**Figure 11:**
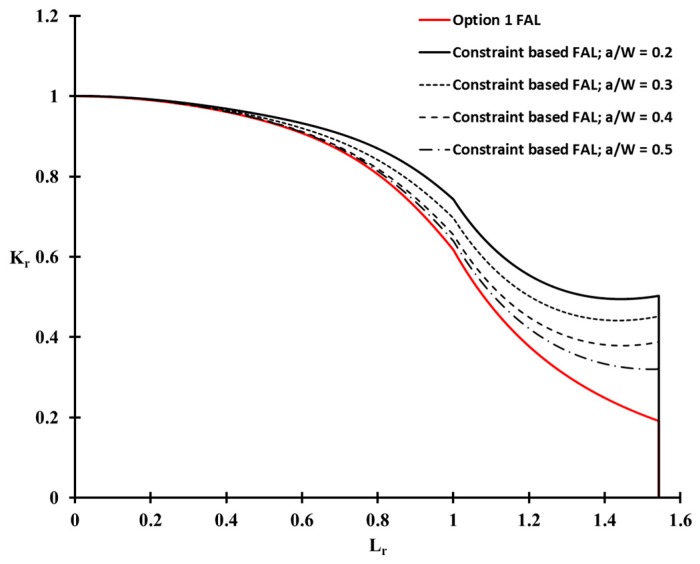
Comparison of the conventional and constraint-based FADs for various crack ligament ratios.

Figure 11 shows the constraint-based FADs for the weldment of Al 5083-O at cryogenic temperature. A significant difference in FADs was observed between the shallow cracked specimens and Option 1. Here, the Option 1 FAD is the conventional FAD based on the BS 7910 [1] procedure, because no correction for the constraint effects is considered. For a deeply cracked specimen, a/W = 0.5, the extent of the correction for the constraint level is not distinct at a low applied load (below $L_{r}$ = 0.6) region. For all a/W ratio cases, however, the constraint-based FADs are extended outside the Option 1 FAD, with increasing applied load (higher than $L_{r}$= 0.6). Therefore, the conventional FAD, such as Option 1 FAD, is significantly more conservative than the constraint-based FAD, which takes the constraint effect into account.

### 5.4. Fracture Assessment for the Welded Joints of Al 5083-O with a Surface Flaw

**Figure 12:**
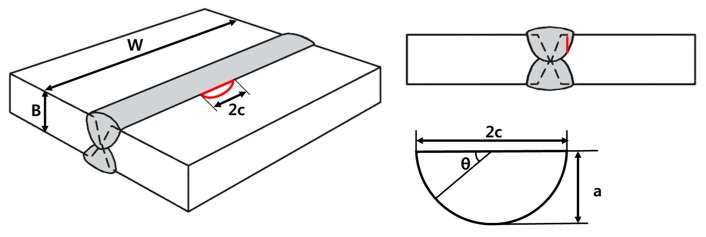
Schematic illustration of the wide plate with a surface crack.

To perform a fracture assessment considering the constraint effect, a wide butt-welded plate with a surface flaw under a tensile load at cryogenic temperature was considered. The thickness, B, and width of the welded plates, W, were 25 and 1000 mm, respectively. Figure 12 presents the surface flaw geometries. The flaw depth and lengths are denoted as “a” and “2c”, respectively. Table 5 lists the various initial surface flaw sizes considered in this study.

In general, the constraint-based fracture assessment requires an evaluation of the constraint parameter, β, as previously defined in Equations (11) and (12), for a structure with a flaw according to BS 7910 [1]. Several studies, however, indicated that the constraint level of the fracture test specimen, and that of a structure with a flaw, typically exhibit a similar tendency. Donoso and Labbe [28] showed that the Q parameter of a pre-cracked cylindrical specimen exhibited similar values to those of the SENB specimens. In addition, Cravero and Ruggieri [29] demonstrated that shallow cracked pipes indicate a significant loss of constraint, and these trends are similar to the SENB specimens [17]. Accordingly, this study examined the correlation of the constraint level between the CT specimen, and the welded plate with a flaw, in terms of the FAD procedure.

As described in Section 2.2, the normalized structural constraint parameter, β, can be described by both the T-stress and Q parameter. For the plate with a surface flaw under a tension load, the $\beta_{T}$ defined in Equation (12) can be expressed as [1]

(17) $$\beta_{T}=\left(1-\alpha^{\prime \prime }\right)\left[X_{0}+X_{1}{\left(\frac{a}{B}\right)}^{2}+X_{2}{\left(\frac{a}{B}\right)}^{4}+X_{3}{\left(\frac{a}{B}\right)}^{6}\right]\;\text{for}\;0\leq a/B\leq 0.8\;\text{and}\;0.2\leq a/c\leq 0.8,$$

where $\alpha^{\prime \prime }=\left(a/B\right)/\left[1+\left(B/c\right)\right]\;$for W≥2(c+B), and $\alpha^{\prime \prime }=2\left(a/B\right)/\left(c/W\right)$ for W<2(c+B). Further details for the polynomial coefficients, $X_{i}\left(i=0,\;1,\;2,\;\mathrm{and}\;3\right)$, are found in Ref. [1].

**Figure 13:**
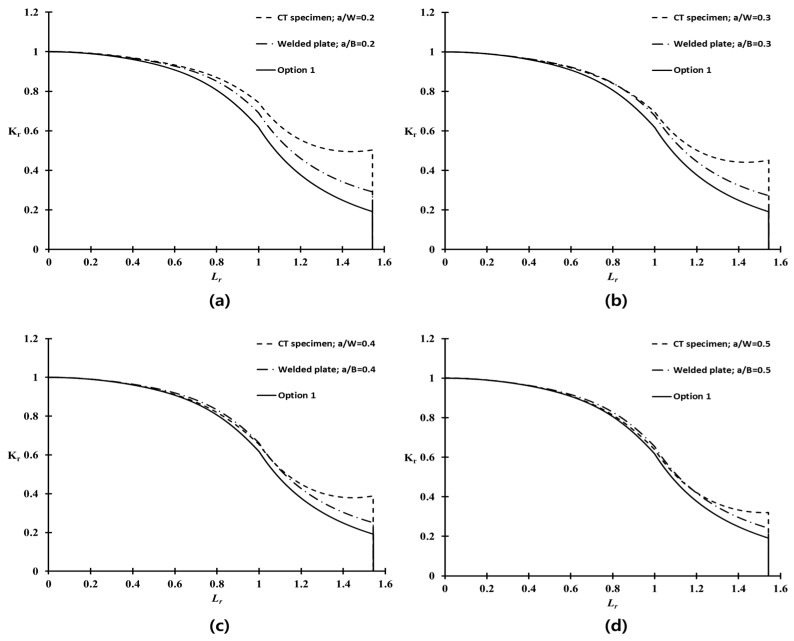
Constraint-based FADs for the welded plate of Al 5083-O with various crack configurations (**a**) a/B = 0.2, (**b**) a/B = 0.3, (**c**) a/B = 0.4, and (**d**) a/B = 0.5.

From Equation (17), $\beta_{T}$ for the welded plate with a surface flaw of Al 5083-O, considered in this work, was calculated corresponding to the various initial flaw size, as shown in Table 5. Figure 13 shows the corresponding constraint-based FAD in terms of $\beta_{T}$ for the deepest point of flaw (θ = 90). In Figure 13, the constraint-based FAD obtained from the CT specimen is also included. The FAL of the welded plate obtained from the $\beta_{T}$ solution using the BS 7910 procedure, and that of the CT specimen based on the $\beta_{Q}$ in the present study, are almost identical for $L_{r}\leq 1$. These results are consistent with the basis that the T-stress and Q parameter provide similar results for $L_{r}\leq 1$ [1]. For $L_{r}>1$, the difference between the FALs of the welded plate and the CT specimen increase gradually, because the effect of plasticity becomes significant at a higher $L_{r}$. Therefore, the BS 7910 procedure recommends the Q parameter based on the elastic–plastic fracture mechanics for $L_{r}\geq 1$. From these results, the constraint-based FADs obtained from the CT specimen could be applied to assess the maximum allowable stress of the welded plate with a surface flaw.

**Figure 14:**
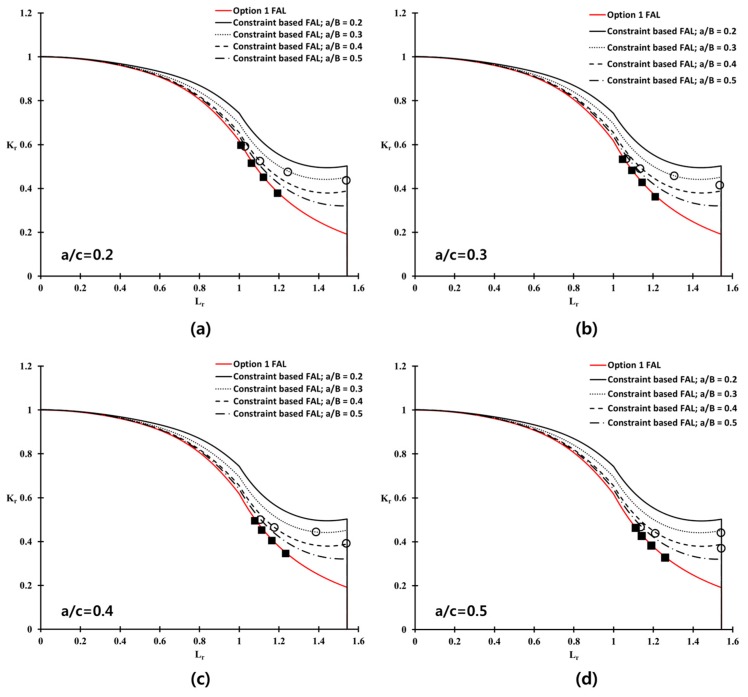
Failure assessment points for the welded plate of Al-5083 based on BS 7910 Option 1 and the constraint-corrected FADs, with different crack configurations (**a**) a/c = 0.2; (**b**) a/c = 0.3; (**c**) a/c = 0.4; and (**d**) a/c = 0.5.

The ratios of the flaw depth to the length of the welded plate between a/c = 0.2, 0.3, 0.4, and 0.5, were considered in this study. Figure 14 presents the failure assessment points from Option 1 and the constraint-based FADs of the welded plate, with respect to the four a/B values. At a given a/c ratio, the failure assessment points from the constraint-based FADs lie on the right side compared to those from Option 1 FAD. This observation is associated with the extended acceptable area of the constraint-based FADs, than that of the Option 1 FAD. As described in Figure 1, the failure assessment point moves gradually toward the right side of the FAL, with increasing applied stress to the structure. In other words, the constraint level corrected by constraint-based FAD shows the higher maximum allowable stress on the welded plate, than that from the Option 1 FAD. 

The FAD method can be used to calculate the residual strength (maximum allowable applied stress). Moreover, if the maximum magnitude of the in-service applied stresses are known, the FAD can be used to calculate the critical flaw size [30].

**Figure 15:**
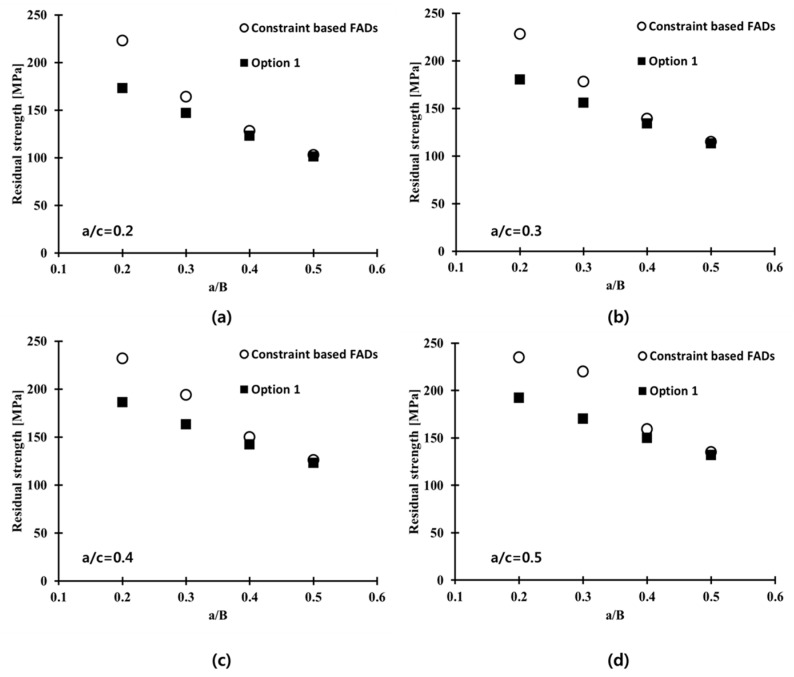
Comparison of the residual strength distribution for the welded plate of Al 5083-O with different crack configurations at cryogenic temperature (**a**) a/c = 0.2; (**b**) a/c = 0.3; (**c**) a/c = 0.4; and (**d**) a/c = 0.5.

Figure 15 shows the residual strength distribution for a welded plate of Al 5083-O with different crack configurations at cryogenic temperature obtained from Option 1 and constraint-based FADs. Here, the residual strength is the maximum allowable stress on the welded plate in the presence of a flaw [30]. For a shallow crack with a/B = 0.2, the residual strength obtained from the constraint-based FAD is higher than that value obtained from Option 1. In other words, the prediction of residual strength by the Option 1 FAD, without a correction for the constraint effect, results in overly conservative estimates, compared to the value obtained from the constraint-based FAD. This was attributed to the low constraint level (low Q parameter) near the crack tip, and the enhanced fracture toughness, due to the loss of constraint near the crack tip in the welded plate [17]. For a deep crack, such as a/B = 0.5, the residual strengths were almost identical regardless of whether Option 1 FAD or constraint-based FAD were used, because the constraint effect is insignificant for deep cracks. Accordingly, the constraint correction near the crack tip employing the constraint-based FAD becomes more important, particularly for shallow cracks. Therefore, a rational fracture design for the welded plate of Al 5083-O with a surface flaw is possible, based on the maximum allowable stress obtained from the constraint-based FAD scheme found in this study.

## 6. Concluding Remarks

This study examined the application capability of the constraint-based fracture assessment for Al 5083-O weldment with a surface flaw at cryogenic temperature. The influence of the constraint level correction on the allowable applied stress was analyzed systematically. The fracture toughness of the base metal, and the weldments for Al 5083-O, were evaluated at room and cryogenic temperatures. In addition, the Q parameters were investigated, based on a series of finite element analyses with various crack configurations, using the CT specimens. Based on the constraint-based FAD methodology, the fracture assessments for the Al 5083-O weldment were conducted to predict the maximum allowable stress in the presence of surface flaws. The residual strengths were compared according to the conventional fracture assessment procedure in BS 7910 Option 1. The major findings from this study are as follows:

- The CTOD values of Al 5083-O at cryogenic temperature were higher than those at room temperature for both the base metal and weldments. In case of the weldment, the average CTOD values were 0.74 mm and 0.91 mm, at room and cryogenic temperatures, respectively. This was attributed to the slower crack opening behavior of the Al 5083-O weldment at cryogenic temperature than room temperature.
- The Q parameter decreases rapidly with increasing applied load. Moreover, the Q parameter values for a shallow crack were calculated to be lower than the values of a deep crack. On the limit load, the Q parameter values of the CT specimen corresponding to a/W = 0.2, 0.3, 0.4, and 0.5 at cryogenic temperature were found to be in the range between −0.55, −0.46, −0.37, and −0.29. No significant temperature effect in the Q parameter was observed in the case of the Al 5083-O weldment.
- Based on the J-Q approach, the critical J integral values of the CT specimen corresponding to a/W = 0.2–0.5 were determined using the modified RKR form. As expected, the critical J integral value were found to be inversely proportional to the Q parameter as the crack length increased, with respect to the ligament of the CT specimen. The α and k values, which are the constants of constraint-based FAD, for the Al 5083-O weldment at cryogenic temperature, were found to be 1.544 and 3.418, respectively. With the J-Q locus calculated in this study for the weldment of Al 5083-O, the fracture toughness with different constraint levels at cryogenic temperature can be estimated readily. In other words, the critical J integral value can be evaluated conveniently using the J-Q locus without requiring further fracture toughness tests.
- The conventional fracture assessment method based on BS 7910 Option 1 FAD produces excessively conservative results if the constraint effect is not considered properly. Based on this study, the maximum allowable stress for the welded plate with a surface flaw of Al 5083-O, which was calculated by the constraint-based FAD, is 29% higher than that obtained from the Option 1 FAD. Therefore, the constraint-based FAD procedure is essential for avoiding overly conservative prediction of the allowable stress from a practical point of view.

## Figures and Tables

**Table 1:** Chemical composition of Al 5083-O.

	Si	Fe	Cu	Mn	Mg	Cr	Zn	Ti	Cr_eq_
Chemical composition (wt. %)	0.07	0.20	0.02	0.60	4.80	0.07	0.01	0.02	0.223

**Table 2:** Welding conditions for the Al 5083-O weldment.

Welding Method	Filler Metal	Groove Shape	Current [A]	Voltage [V]	Speed [cm/min]
MIG welding	ER5183	Double V	550	30	36

**Table 3:** Mechanical properties of the base metal and weldments.

Material	Temp.	Yield Strength [MPa]	Tensile Strength [MPa]	Elastic Modulus [GPa]	Elongation [%]	Strain Hardening Exponent	Material Constant
Base metal	Room	166	326	61	23	7	0.735
	Cryogenic	189	373	70	26	7.13	0.741
Weld metal	Room	157	307	61	20	6.83	0.777
	Cryogenic	175	365	75	23	6.43	0.857

**Table 4:** Fracture toughness of the base metal and weldments for Al 5083-O.

Specimen (a/W = 0.5)	Temp.	CTOD (avg.) [mm]	Max. Load [kN]	CMOD [mm]
Base metal	Room	0.70	46	2.50
	Cryogenic	0.83	57	3.03
Weld metal	Room	0.74	44	2.88
	Cryogenic	0.91	52	3.35

**Table 5:** Initial surface flaw size of the welded plate assumed in this study.

Flaw Type	a [mm]	2c [mm]	a/B	a/c
5 × 50	5	50	0.2	0.2
7.5 × 60	7	60	0.3	0.25
10 × 100	10	100	0.4	0.2
12.5 × 100	12.5	100	0.5	0.25
